# Impact of discontinuing automatic reflex urine culture after urinalysis: a diagnostic and antibiotic stewardship initiative

**DOI:** 10.3389/fcimb.2025.1572936

**Published:** 2025-10-10

**Authors:** Blaine Berger, Janell Lukey, Chetan Jinadatha, Dhammika H. Navarathna

**Affiliations:** ^1^ Department of Pathology and Laboratory Medicine Services, Central Texas Veterans Health Care System, Temple, TX, United States; ^2^ Department of Pathology and Laboratory Medicine, Baylor Scott & White Medical Center, Temple, TX, United States; ^3^ Department of Medicine, Central Texas Veterans Health Care System, Temple, TX, United States; ^4^ Department of Medical Education, School of Medicine, Texas A&M University, Bryan, TX, United States

**Keywords:** asymptomatic bacteriuria, urinalysis reflex, UTI, uropathogen, urine culture

## Abstract

**Introduction:**

This study aims to assess the impact of discontinuing reflex urine cultures based on urinalysis results (positive for nitrates and/or leukocyte esterase) on diagnostics,antibiotic usage, and laboratory efficiency at the Central Texas Veterans’ HealthCare System (CTVHCS). It seeks to evaluate whether stopping reflex testing reducesunnecessary antibiotic use, enhances antibiotic stewardship, and improves theprocessing of clinically relevant specimens.

**Methods:**

A 6-year retrospective analysis was conducted, comparing data from 3 years before and 3 years after the 2018 policy change, which discontinued reflex urine culture unless specifically requested by healthcare providers suspecting urinary tract infection (UTI) symptoms. The study analyzed the number of processed urine cultures, positivity for uropathogens, and antibiotic usage trends before and after the policy change.

**Results:**

The policy change resulted in a significant reduction in processed urine cultures. There was also a notable decrease in ciprofloxacin usage and an increase in the use of nitrofurantoin,indicating a shift towards narrower-spectrum antibiotics.

**Discussion:**

Stopping reflex testing reduced the lab burden by focusing on clinically relevant cases of UTIs and supported improved antibiotic stewardship. This enabled healthcare providers toselectively order culture and sensitivity, targeting true UTIs.

## 1 Introduction

Urinary tract infections (UTIs) due to bacterial pathogens are a very common global issue that draws more than 10 million physicians’ visits and approximately 2 million emergency department visits annually (Schappert and Rechtsteiner, 2011). The most common organisms causing UTIs are *Escherichia coli*, *Enterococcus*, *Klebsiella*, *Enterobacter*, and *Proteus* spp., along with *Staphylococcus saprophyticus* and *Streptococcus agalactiae* (GBS) (Yarbrough; Flores-Mireles et al., 2015).

Early detection of UTI and its etiology along with antibiotic susceptibility helps providers with better patient management. The appropriate method to diagnose UTI is by performing laboratory culture and sensitivity testing of urine samples submitted from patients exhibiting UTI symptoms such as dysuria, urgency, and frequency, which may be associated with systemic symptoms such as fever and chills (Hooton and Stamm, 1997). Laboratory practices and algorithms largely vary across the country. In most hospitals in the USA, after a provider orders a urinalysis (UA) for non-UTI-related conditions, the urine is often automatically reflexed for culture and sensitivity based on hospital-specific criteria (Advani et al., 2019). This usually occurs when abnormalities are detected in leukocyte esterase, nitrite, or white blood cell (WBC) count, to rule out a UTI. There are no prescribed standard/s for urine reflex testing, and each individual healthcare facility makes their own policy for what is the best practice for their patient population and their available laboratory resources. Sometimes, the local laboratory practices conflict with diagnostic and antibiotic stewardship practices at the facility and successful diagnostic stewardship interventions are based on local patient populations and individual patient-based interventions (Advani and Vaughn, 2021).

The Veterans Affairs (VA) healthcare systems including our institution, similar to many other institutions, historically adopted UA with reflex to culture as a strategy to improve the diagnosis of UTIs. This approach was reported to be useful in emergency departments (EDs), where rapid decision-making is critical, making early urine sample collection and processing advantageous (Chironda et al., 2014). Additionally, in adult intensive care units (ICUs), reflex urine culturing has been associated with a reduction in the number of urine cultures ordered, although its impact in other clinical areas remains uncertain (Sarg et al., 2016). Despite limited setting-specific data and variable patient populations, many healthcare systems continue to rely on the UA reflex to culture method as a standard diagnostic protocol (Advani and Vaughn, 2021). Our institution undertook a process improvement policy change at reducing inappropriate treatment of asymptomatic bacteriuria focusing on optimizing urine test utilization. Our hospital has a strong antibiotic stewardship working group composed of infectious disease (ID) physicians, clinical pharmacists, an infection preventionist, and a microbiologist. ID specialists raised the issue of indiscriminate antibiotics used for asymptomatic bacteriuria and suggested the possibility of UA reflex hard stop. As our pregnant patient population is very low, the lab agreed to pursue this. Our microbiology lab classifications and workup of potential uropathogens in nonsterile urines are based on the Clinical Microbiology Procedures Handbook. Gram-negative Bacilli, *Staphylococcus aureus*, *Enterococcus* spp., beta-hemolytic *Streptococcus, Neisseria gonorrhoeae*, and yeasts are considered uropathogens (Yarbrough).

Here, we describe the effect of policy change to discontinue reflex urine testing from all positive UA samples in our facility. Five years post policy change, we examined our retrospective data to understand the impact of our process improvement measures on both diagnostic and antibiotic stewardship fronts.

## 2 Material and methods

### 2.1 Facility

The Central Texas Veterans’ Health Care System (CTVHCS) provides healthcare services at 11 locations serving central Texas. Our hospitals are the Olin E. Teague Veterans Medical Center in Temple and the Doris Miller VA Medical Center in Waco. We also operate a stand-alone multispecialty clinic in Austin, and nine community-based outpatient clinics in various small cities in central Texas locality. We serve approximately 250,000 patients in 39 counties in central Texas.

### 2.2 Study material and samples

#### 2.2.1 Sample collection and processing

Midstream, clean-catch urine specimens were collected in sterile, wide-mouthed, leak-proof containers according to standard microbiological procedures. Samples were transported to the laboratory within 2 h of collection, or stored at 4 °C and processed within 24 h when immediate inoculation was not feasible.

#### 2.2.2 Primary culture

Each urine specimen was mixed thoroughly, and 1 µL of uncentrifuged urine was inoculated onto UriSelect™ chromogenic agar (Bio-Rad, France) using a calibrated loop. Plates were incubated aerobically at 35 ± 2 °C for 18–24 h. Colony counts were recorded as colony-forming units per milliliter (CFU/mL) and interpreted according to established diagnostic thresholds.

#### 2.2.3 Colony morphology and preliminary identification

Chromogenic reactions on UriSelect agar were observed for preliminary differentiation of bacterial species based on colony color and morphology (e.g., *Escherichia coli*—pink to burgundy colonies; *Enterococcus* spp.—turquoise colonies; *Klebsiella*, *Enterobacter*, and *Citrobacter*—blue colonies; *Proteus* spp.—brown colonies). Mixed cultures were noted and isolated further as required.

#### 2.2.4 Definitive identification by MALDI-TOF MS

Representative colonies were subjected to species-level identification using MALDI-TOF mass spectrometry (MS Prime system) (bioMérieux, France).

#### 2.2.5 Antimicrobial susceptibility testing

Isolates were subjected to antimicrobial susceptibility testing (AST) using the Vitek 2 system (bioMérieux, France). A 0.5 McFarland standard suspension was prepared in sterile saline and inoculated into appropriate Vitek 2 AST cards (e.g., GN or GP panels depending on organism type).

All urine culture testing is done at the central microbiology laboratory located at our main campus in Temple, TX. Specimen requirement by the lab is clean-catch or catheterized urine samples to be transported to the laboratory as soon as possible. If a delay in transport is expected, the specimen may be transferred to a preservative tube that may be held at room temperature or refrigerated for up to 48 h. Urine specimens are held refrigerated for 2 days after setting up for culture and susceptibility testing. A urine culture is considered positive if it grows up to two organisms >10 colonies in our lab using 1 μL of inoculating loop (1×10^4^/mL). For freshly voided urine, a culture of ≥100 CFU (1×10^5^/mL) from a single organism type is considered the cutoff. If the CFU range is between 10 and 100, evaluation will be based on the specimen type and clinical status. Samples will not be worked up for less than 10 CFUs except for cystoscopic or kidney/suprapubic aspirates. As part of continuous process improvement, in August 2014, we switched to nonselective chromogenic culture medium from a conventional culture method to process urine samples. This was considered more time-saving with reliable detection, easy enumeration, presumptive identification, and easy recognition of mixed cultures (Hengstler et al., 1997).

CTVHCS uses the Sysmex UN-series, an automated UA system for screening and initial clinical workup of a variety of renal and lower urinary tract disorders. From late 2013 to mid-August 2018, our central lab located at the Olin E. Teague Veterans Medical Center in Temple, TX received urine specimens showing positive UA results for nitrates, and/or leukocyte esterase, which were subsequently subjected to reflex culture and sensitivity. Negative reflex cultures would be reported with “no significant bacterial growth” while positive cultures would be reported with ID and sensitivity with a comment entered as “CLO” (culture indicated by UA, laboratory ordered) on the laboratory order. In 2018, reflex testing was abandoned in favor of provider-requested urine culture. Since 6 August 2018, the hospital’s microbiology lab has ceased performing reflex urine cultures. According to the guidelines of the United States Preventive Services Task Force, screening for asymptomatic bacteriuria is only recommended for pregnant women (Henderson and Bean, 2019). This recommendation is mirrored by the latest IDSA Clinical Practice Guideline for the Management of Asymptomatic Bacteriuria except in older persons residing in long-term care facilities (Nicolle et al., 2019). All our pregnant women and veterans in long-term care are examined by the women’s clinic or their providers who directly ordered urine culture and sensitivity testing. Consequently, leukocyte esterase- and nitrite-positive UA results were no longer reflexively sent for culture and sensitivity testing, and the “CLO” comment was not included in patient charts. A laboratory memo was distributed to all providers to notify them of the changes made, informing them not to assume that UA will automatically reflex for future cultures and emphasizing the requirement to place a separate culture order for suspected UTIs.

### 2.3 Data collection

**Figure 1:**
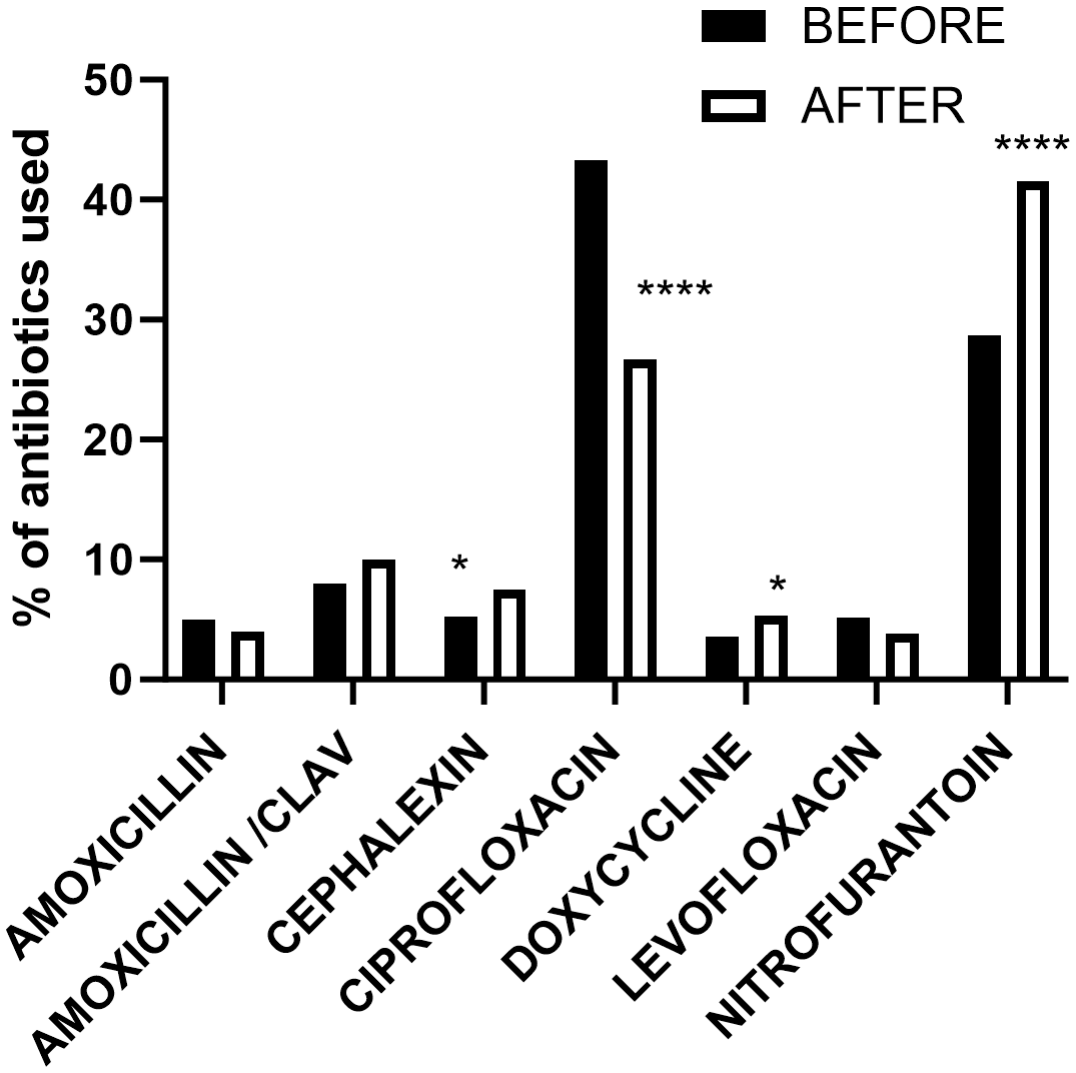
Patient’s records of administered or prescribed antibiotics dose regimen per patient in the 3 years preceding and following the implementation of hard stop urine reflex policy. *p < 0.05; ****p < 0.0001.

Using the patient electronic medical record system and TheraDoc (Premier, Inc., Charlotte, NC), an electronic clinical surveillance system, positive urine culture results were collected. We examined retrospective urine culture results for the 3 years before and 3 years after July 2018, which marks an inflection point where UA reflex to culture ceased. To analyze the impact on antibiotic utilization, we collected data on patients diagnosed as having UTI and treated with antibiotics (dose per patient) 3 years before and 3 years after July 2018. The hospital antibiotic usage percentages for UTI therapy before and after the policy decision are depicted in **Figure 1**.

### 2.4 Statistics analysis

Analysis of contingency tables with major uropathogens (**Table 1**) was performed by using the chi-square test and antibiotic usage trend using Fisher’s exact test in GraphPad Prism version 10.1.2 for Windows (GraphPad Software, San Diego, CA; www.graphpad.com).

**Table 1:** Total number of real uropathogens reported from the urine culture and sensitivity (C&S) workup in 3 years before and 3 years after the hard stop on urinalysis reflex to culture and percent reduction.

Uropathogen	# Pre-decision urine C&S	# Post-decision urine C&S	% Reduction
*S.agalactia*	768	187	76
*S.aureus*	309	92	70
*E faecalis*	1840	742	60
*E coli*	4794	2310	52
*K. pneumoniae*	968	666	31
*P mirabilis*	564	300	47

# is shorten abbreviation for number.

## 3 Results

### 3.1 Comparison of all urine cultures ordered pre and post 3 years from stop reflex decision

We examined the total number of urine culture orders received at the microbiology lab for culture and sensitivity for 3 years before and 3 years after July 2018. Three years before the policy change, our microbiology lab processed 47,288 total urine samples. In the subsequent 3 years following the intervention, the number of samples processed dramatically reduced to 27,366 urine samples, which is a 42% decline.

### 3.2 Comparison of all positive urine cultures reported pre and post 3 months from stop reflex decision

First, we examined all reported positive urine cultures 3 months pre and post decision date, spanning a total of 6 months (**Table 2**). We noticed a huge reduction in the number of urine cultures processed in our laboratory. Our lab reported 1,511 positive urine cultures during the 3 months immediately before the policy was changed to stop reflex-based UA reports. Three months after implementing the new policy, the lab only reported 409 positive urine cultures. Reports of positive cultures from normal flora in the urine show that the *Lactobacillus* and *Streptococcus* viridans group had a dramatic reduction from 47 and 41 to 9 and 4, respectively (**Table 2**). In addition, there were 16 candida and non-candida yeast reported in the 3 months pre-decision period, while only 2 were reported after the policy was changed. We also noticed a significant reduction in the major uropathogens (*p* < 0.05) in terms of *E. coli*, *K. pneumoniae*, *Proteus* spp., *Enterococcus* spp., coagulase-positive *Staphylococcus*, and group B streptococcus (**Table 2**).

**Table 2:** Total number of each organism reported from the culture and sensitivity (C&S) workup in 3 months before and after the hard stop on urinalysis reflex.

UTI organism	# Reported pre decision on C&S	# Reported post decision on C&S
E. coli *	597	152
E. faecalis *	185	56
K. pneumoniae *	152	46
S. agalactiae *	92	21
S. epidermidis **	88	18
P. mirabilis *	59	26
Lactobacillus **	47	9
S. viridans **	41	4
S. anginosus **	32	16
E. cloacae *	25	13
S. aureus *	23	11
C. koseri *	22	4
K. oxytoca *	21	5
P. aeruginosa *	13	8
S. marcescens *	12	2
S. haemolyticus **	12	2
K. aerogenes *	8	3
C. braakii **	7	0
P. stuartii **	7	2
M. morganii *	6	5
S. saprophyticus **	5	0
S. alactolytus **	4	0
S. mutans ***	4	0
S. simulans ***	4	0
E. gallinarum ***	4	0
Corynebacterium **	4	0
P. rettgeri **	4	1
R. planticola *	4	0
S. gallolyticus **	4	1
S. warneri ***	3	0
A. baumannii *	3	0
E. faecium *	3	2
C. glabrata *	8	1
C. albicans *	8	0
C. tropicalis *	0	1

Uropathogen* Opportunistic** Non-pathogen***.

### 3.3 Comparison of major uropathogens reported pre and post 3 years from stop reflex decision

After our initial pan comparison of all UTI reports for the 6-month time frame, we examined the trends of major pathogens of concern (Flores-Mireles et al., 2015) by extending the 3 years pre and 3 years post decision to stop reflexing (**Table 1**) from the total cultures processed and mentioned in section 3.1. As expected, we found a significant reduction of all listed pathogens (*p* < 0.0001). *S. agalactiae* (GBS) was the least reported, with a reduction of 76% of cases from pre to post intervention. *K. pneumoniae*, being a real uropathogen, had a 31% reduction in reported culture positivity.

#### 3.3.1 Antibiotic utilization trend before and after policy implementation

We identified 1,249 patient records (dose regimen/patient used for UTI only) with administered or prescribed antibiotics in the 3 years preceding policy implementation and 1,633 patient records post-implementation as shown in **Table 3**. Analyzing the data (**Figure 1**) using Fisher’s exact test, we observed a significant reduction in ciprofloxacin usage (*p* < 0.0001) post-implementation, while nitrofurantoin usage increased significantly (*p* < 0.0001). Additionally, cephalexin and doxycycline usage showed significant increases (*p* < 0.05) after policy implementation.

**Table 3:** Total antibiotics classes (dose regimen/patient) prescribed 3 years before and 3 years after discontinuing reflex testing (dose regimen/patient).

Antibiotic name	# Used for UTI before* decision	# Used for UTI after* decision
AMOXICILLIN	65	69
AMOXICILLIN/CLAV	104	171
CEPHALEXIN	66	123
CIPROFLOXACIN	541	435
DOXYCYCLINE	45	87
LEVOFLOXACIN	65	62
NITROFURANTOIN	358	679
FOSFOMYCIN	1	3
CEFTRIAXONE	4	4
Total # of Antibiotics used for	1249	1633

# is shorten abbreviation for number. * is for discontinuing reflex testing.

## 4 Discussion

UA is often considered an unreliable tool for diagnosing UTIs because of its low positive predictive value (PPV) when compared to urine culture. For instance, the PPV of pyuria in identifying culture-positive infections has been reported to be as low as 4% and up to 32% in various studies (Humphries and Dien Bard, 2016). The absence of standardized criteria for reflexive urine culture testing has recently become a subject of investigation. UA is one of the oldest diagnostic tests in medicine, and today, many laboratories utilize UA as a preliminary screening method to decide whether further bacterial culture is necessary. While reflex urine culture is now a common practice, there is a lack of evidence-based guidelines to define optimal criteria and workflows for these procedures (Chambliss and Van, 2022). Prior research has highlighted similar challenges, noting that inappropriate treatment of asymptomatic bacteriuria is often linked to the overuse of UA reflex to urine cultures. The findings revealed that halting reflex urine culture practices significantly reduced the number of cultures performed and showed a tendency toward lower antibiotic usage (Dietz et al., 2016). The purpose of this study was to evaluate the effects of discontinuing reflex urine cultures, and these results align with previous studies on the subject (Dietz et al., 2016; Chambliss and Van, 2022).

Uropathogens in urine culture may not be an indication of UTI in many cases. In ICU trauma patients, Stovall et al. found that a negative UA rules out a catheter-associated UTI in virtually all cases due to its high negative predictive value and sensitivity of 100% (Stovall et al., 2013). A positive UA was defined as positive leukocyte esterase, positive nitrite, WBC > 10/high-power field, or the presence of bacteria. A positive urine culture was defined as growth of ≥10 (5) CFU of an organism irrespective of the UA result or ≥10 (3) CFU in the setting of a positive UA. A UTI was defined as positive urine culture without an alternative cause for the fever (Stovall et al., 2013). Cultures performed without additional diagnostics tests that aid in the correct clinical interpretation of urine cultures is a common practice. A study of isolated urine cultures in 2009–2013 found that 20.2% of urine cultures were performed without a UA or urine microscopy (Carlson et al., 2017). This number may vary from institution to institution based on protocols, but this can add up to a large volume of urine cultures, especially at larger hospitals and healthcare systems. According to Stovall et al (Stovall et al., 2013), many of these urine cultures could be prevented by implementing a policy of not performing urine cultures on a patient with a negative UA because even in cases of positive urine cultures, a negative UA ruled out catheter-associated UTI in patients with a fever. We find that this practice of discontinuing reflex of positive UA to culture may further lead to a significant reduction in urine culture volume, with the most benefit seen in larger institutions with higher volumes of patients and specimens. This decreases the burden on the lab and allows the staff to focus on processing more critical specimens instead of working up cultures that may not be clinically relevant. A positive urine culture may extend a patient’s stay and cause them to receive additional treatments and is also a detriment to antibiotic stewardship efforts. In cases where these positive urine cultures likely do not represent an underlying UTI, the patient may receive unnecessary antibiotic treatment and have a prolonged ICU or hospital stay (Grein et al., 2016). This leads to increased chances of patients developing healthcare-related infections or other harm, increasing patient morbidity and mortality. Unnecessary antibiotics can promote the development of antibiotic-resistant bacteria. These antibiotic-resistant bacteria can pose significant harm to patients and become challenging to treat, increasing patient morbidity and mortality (Ventola, 2015). Although targeted antibiotics for UTIs were used more frequently than non-specific antibiotics (at the discretion of the provider, as is common practice at our institution), some providers treated UTIs without ordering a urine culture. This practice may have contributed to the increased total number of antibiotics used (shown in **Table 3**), which is a limitation of this study. Our electronic health record system cannot specifically isolate cases where a urine culture and sensitivity was performed for UTI diagnosis and treated accordingly. Once we stopped reflexing, we only reported antibiotic sensitivity results from the patients who had orders. In addition, the annual hospital antibiogram, which includes ESBL prevalence, provides good insight into locally resistant trends based on those results for individual physicians to make a therapeutic choice. Therefore, targeted therapy and empirical therapy were both impacted by the policy of stopping UA reflexes to culture, and we believe that the trend shift in antibiotic use occurred because of this.

In addition to the financial burden to the patient and the hospital system, another considerable factor is that the hospitals are not reimbursed for treatment of catheter-associated UTIs. Therefore, it is very important for patient safety that results are accurate and reliable in these diagnoses (Morgan et al., 2012).

Larger healthcare systems are most affected by these inefficiencies and will see the greatest benefit from policy changes to reduce unnecessary urine cultures. Urine cultures can be falsely positive due to improper collection, improper handling, improper storage conditions, and contamination. Therefore, in addition to revisiting reflex criteria, as recommended by Stovall et al (Stovall et al., 2013), UA of suspected culture-positive patients will also have an added benefit. However, the latter option comes at an additional cost. Standardizing these procedures and ensuring proper quality control help increase efficiency and decrease false positives. The significant increase in nitrofurantoin usage post-implementation serves as a clear indication of the successful introduction of narrower-spectrum antibiotics, which aligns with the principles of antibiotic stewardship. This shift towards more targeted antibiotic therapy reflects a conscious effort to minimize the use of broad-spectrum antibiotics and mitigate the risk of antibiotic resistance. In addition to the seven common antibiotics we used for this study (**Figure 1**), trimethoprim/sulfamethoxazole and ceftriaxone (included in **Table 3**) are commonly used for simple UTI. In complicated UTI or kidney infection, fluoroquinolone is used if there are no other treatment options (Jancel and Dudas, 2002). Another limitation in our data is not including empirical use of antibiotics before the culture results were available. Another limitation was that our electronic data system could not retrieve purely culture-based antibiotics therapy data. As a result, we computed total antibiotic dose data per UTI case diagnosed through cultures and symptomatic evaluations.

Moreover, the implementation of selective reporting of antibiotic sensitivity due to formulary restriction and the latest CLSI updates may have played a role in reducing the use of targeted antibiotics such as ciprofloxacin. By providing healthcare providers with more precise information on antibiotic susceptibility, selective reporting promotes the use of antibiotics that are most effective against specific pathogens, thereby optimizing targeted antibiotic therapy and reducing unnecessary antibiotic use by skipping asymptomatic bacteriuria. We believe that our report will help other organizations to revisit their policies, in turn aiding in cost cutting, improving patient safety, and promoting antibiotic stewardship. Urinary cultures should only be performed when there is a high clinical suspicion for infection rather than a reflex testing of every abnormal UA.

## Data Availability

The original contributions presented in the study are included in the article/supplementary material. Further inquiries can be directed to the corresponding author.
